# Supplementary material for: Age differences in the conceptualization and experience of curiosity: A qualitative study
Source: PLoS One. 2026 May 20;21(5):e0345902. doi: 10.1371/journal.pone.0345902 (PMC13189317; doi:10.1371/journal.pone.0345902)
Supplement: S1 Table — Note. For all chi-square values, degrees of freedom (df) = 1. Under younger and older adult sample sections, numbers in parentheses indicate total percentages for corresponding code frequencies. Absence of χ2 and 95% CI values indicate that frequencies across both samples were too low for accurate comparisons. Benjamini-Hochberg (BH) corrected p-values are also included to correct for multiple comparisons. * p < .05. ** p < .01. *** p ≤ .001. (DOCX) [file pone.0345902.s001.docx]

**S1 Table. Chi-Square Tests for Definition Question Codes.**

|  | **Sample** | |  |  |  |  |  |
| --- | --- | --- | --- | --- | --- | --- | --- |
| **Coding Categories** | **Younger Adults** | **Older Adults** | ***χ^2^*** | ***p-value*** | **BH *p-value*** | **Cramér’s V** | **95% CI** |
| Joyous exploration | 68 (90.70%) | 77 (96.30%) | 1.99 | .16 | .26 | .11 | [-0.13, 0.02] |
| Deprivation sensitivity | 34 (45.30%) | 8 (10.00%) | 24.46 | < .001*** | < .001*** | .40 | [0.22, 0.48] |
| Stress tolerance | 1 (1.30%) | 5 (6.20%) | - | .21 | .32 | - | - |
| Social curiosity | 18 (24.00%) | 35 (43.80%) | 6.71 | .009** | .05 | .21 | [-0.34, -0.05] |
| Thrill seeking | 8 (10.70%) | 1 (1.30%) | 6.28 | .012* | .05 | .20 | [0.02, 0.17] |
| Intrapersonal curiosity | 6 (8.00%) | 3 (3.80%) | 1.28 | .26 | .34 | .09 | [-0.03, 0.12] |
| Miscellaneous | 38 (50.70%) | 40 (50.00%) | 0.01 | .93 | 1.00 | .01 | [-0.15, 0.16] |
| Future thinking | 4 (5.30%) | 4 (5.00%) | - | 1.00 | 1.00 | - | - |
| Centrality | 8 (10.70%) | 2 (2.50%) | 4.28 | .039* | .12 | .17 | [0.00, 0.16] |
| Individual differences | 18 (24.00%) | 28 (35.00%) | 2.24 | .13 | .27 | .12 | [-0.25, 0.03] |
| Innovation | 9 (12.00%) | 4 (5.00%) | 2.47 | .12 | .27 | .13 | [-0.02, 0.16] |
| Other | 5 (6.70%) | 6 (7.50%) | 0.04 | .84 | 1.00 | .02 | [-0.09, 0.07] |

*Note.* For all chi-square values, degrees of freedom (*df*) = 1. Under younger and older adult sample sections, numbers in parentheses indicate total percentages for corresponding code frequencies. Absence of *χ^2^* and 95% CI values indicate that frequencies across both samples were too low for accurate comparisons. Benjamini-Hochberg (BH) corrected p-values are also included to correct for multiple comparisons.

** p < .05*. ** *p < .01*. *** *p* ≤ *.001*.
