# Supplementary material for: Age differences in the conceptualization and experience of curiosity: A qualitative study
Source: PLoS One. 2026 May 20;21(5):e0345902. doi: 10.1371/journal.pone.0345902 (PMC13189317; doi:10.1371/journal.pone.0345902)
Supplement: S2 Table — Note. For all chi-square values, degrees of freedom (df) = 1. Under younger and older adult sample sections, numbers in parentheses indicate total percentages for corresponding code frequencies. Absence of χ2 and 95% CI values indicate that frequencies across both samples were too low for accurate comparisons. Benjamini-Hochberg (BH) corrected p-values are also included to correct for multiple comparisons. * p < .05. ** p < .01. *** p ≤ .001. (DOCX) [file pone.0345902.s002.docx]

**S2 Table. Chi-Square Tests for Valence Question Codes.**

|  | **Sample** | |  |  |  |  |  |
| --- | --- | --- | --- | --- | --- | --- | --- |
| **Coding Categories** | **Younger Adults** | **Older Adults** | ***χ^2^*** | ***p-value*** | **BH *p-value*** | **Cramér’s V** | **Confidence Interval** |
| Positive trait | 55 (73.30%) | 73 (91.20%) | 8.64 | .003** | .01* | .24 | [-0.29, -0.06] |
| Negative trait | 1 (1.30%) | 1 (1.30%) | - | 1.00 | 1.00 | - | - |
| In moderation | 33 (44.00%) | 14 (17.50%) | 12.87 | < .001*** | .002** | .29 | [0.13, 0.41] |
| Motivated learning | 38 (50.70%) | 37 (46.20%) | 0.30 | .58 | .69 | .04 | [-0.11, 0.20] |
| Critical process | 19 (25.30%) | 14 (17.50%) | 1.42 | .23 | .34 | .10 | [-0.05, 0.21] |
| Novelty-driven | 28 (37.30%) | 14 (17.50%) | 7.71 | .006** | .01* | .22 | [0.06, 0.34] |
| Advance knowledge | 28 (37.30%) | 25 (31.20%) | 0.64 | .43 | .55 | .06 | [-0.09, 0.21] |
| Personal growth | 30 (40.00%) | 13 (16.20%) | 10.89 | .001*** | .004** | .27 | [0.10, 0.38] |
| Harmful | 37 (49.30%) | 17 (21.20%) | 13.45 | < .001*** | .002** | .30 | [0.14, 0.43] |
| Sincerity | 8 (10.70%) | 18 (22.50%) | 3.88 | .049* | .11 | .16 | [-0.23, -0.003] |
| Individual differences | 15 (20.00%) | 18 (22.50%) | 0.14 | .70 | .76 | .03 | [-0.15, 0.10] |
| Centrality | 9 (12.00%) | 4 (5.00%) | 2.47 | .12 | .19 | .13 | [-0.02, 0.16] |
| Miscellaneous | 8 (10.70%) | 17 (21.20%) | 3.21 | .07 | .14 | .14 | [-0.22, 0.01] |

*Note.* For all chi-square values, degrees of freedom (*df*) = 1. Under younger and older adult sample sections, numbers in parentheses indicate total percentages for corresponding code frequencies. Absence of *χ^2^* and 95% CI values indicate that frequencies across both samples were too low for accurate comparisons. Benjamini-Hochberg (BH) corrected p-values are also included to correct for multiple comparisons.

** p < .05*. ** *p < .01*. *** *p* ≤ *.001*.
