# Supplementary material for: Age differences in the conceptualization and experience of curiosity: A qualitative study
Source: PLoS One. 2026 May 20;21(5):e0345902. doi: 10.1371/journal.pone.0345902 (PMC13189317; doi:10.1371/journal.pone.0345902)
Supplement: S1 Text — (DOCX) [file pone.0345902.s003.docx]

**S1 Text. Category Code Combinations.**

Across both samples, we were interested in identifying patterns among the different possible combinations of initial and rationale responses for the *Valence Question*. We examined the coding category combinations (i.e., initial response and corresponding rationale) that were most frequent among both samples. Descriptive frequencies below and within tables only contain frequency combinations present in ≥ 10% of responses as these were determined to be the most frequent combinations. As there was minimal overlap in the types of combinations that were present between samples, we present descriptive results for each sample separately, listed in order of prevalence.

***Younger Adult Coding Category Combinations***

The most prevalent coding category combination (42.67%; see S3 Table; S1 Figure) was that of curiosity being a *positive trait*, with the rationale being *motivated learning*. Participants expressed that the motivation or general desire to gain greater knowledge is a positive or desirable characteristic. One participant wrote:

I think it [curiosity] is a positive trait because curiosity helps you learn a lot more. If youre constantly curious about things you will most likely do research on that thing to get to the bottom of it. Knowledge really is power so it's important to constantly search for knowledge, that is why I think curiosity is key.

The next most prevalent (41.33%) combination of responses was an initial response of *in moderation* and a rationale of *harmful*. Participants noted that curiosity may be positive or negative depending on how it is practiced. Specifically, one must practice curiosity in moderation, or within limits, to avoid any undue harm or negative outcomes that excessive curiosity could cause. For example: “I think it [curiosity] is both equally positive and negative depending on the context....However, if one is curious about bad subjects, or some more controversial subjects, it can be a bad thing as it can lead to possible harmful and dangerous experiences.”

In a third of responses (33.33%), curiosity was again noted as a desirable or *positive trait* as it allows for *personal growth* or the development of new skills, behaviours, or understanding about oneself. One respondent indicated that curiosity is “definitely positive....curiosity leads to new discoveries that allow you to become a better version of yourself.”

Curiosity was also considered a *positive trait* as it provides opportunities to *advance knowledge*. In just under a third (30.67%) of responses, participants regarded curiosity positively as it provides opportunities to adapt, to innovate, and to express oneself creatively:

Curiosity is generally a positive trait because it drives...innovation...It encourages individuals to...challenge assumptions, leading to deeper understanding and creativity. Curiosity fuels scientific discoveries, technological advancements, and artistic expression, making it essential for progress in various fields.

Younger adults also believed curiosity to be a *positive trait* due to its link to learning and experiencing new things (i.e., *novelty driven*; 30.67%). For example, one younger adult stated: “Curiosity is a positive trait as curiosity is the drive to...discover new things. If not for curiosity there would be no learning and no new experiences in the world.”

In about a quarter of responses (24.00%) curiosity was described as a *positive trait*, with the caveat that it could be *harmful*, or cause undesirable outcomes if practiced without caution. Specifically, one person noted: “I see curiosity as a largely positive trait....However, in some cases, unchecked curiosity can lead to unnecessary risks or distractions.”

In over a fifth of responses (21.33%), younger adults indicated that curiosity is a *positive trait* because it is a *critical process*. Curiosity’s valence (i.e., as a positive or negative trait) was described in light of its ability to prompt one to ask questions and be inquisitive, which was viewed as desirable. For example: “I believe [curiosity is] a positive trait....Without curiosity people would lack the ambition to find answers for questions they do not [have] the answer to.”

In exactly a fifth (20.00%) of responses, *in moderation* and *motivated learning* were paired together. Overall, participants indicated that while curiosity can be considered both a positive and negative trait, when elaborating on the positive nature of curiosity, participants noted that it allows for greater learning in a general capacity: “I personally believe it [curiosity] to be more of a positive trait as it makes individuals want to learn more and get a better understanding of certain situations....Although it can have its negative side aswell…”

Similarly, in less that a fifth of responses (17.33%), participants indicated that curiosity can be both positive and negative (i.e., *in moderation*), and when elaborating on the desirable nature of curiosity, younger adults expressed how it allows for one to develop skills or grow as a person (i.e., *personal growth*). This is displayed in the following open-ended response:

Curiosity is generally a positive trait, as it drives...personal growth....Curiosity also contributes to personal and social development. It promotes open-mindedness, empathy, and a willingness to understand different cultures, viewpoints, and lifestyles. This can lead to stronger relationships and a more inclusive society....However, curiosity can have negative aspects…

Younger adults also expressed curiosity to be both a positive and negative trait (i.e., *in moderation*) as it allows for one to *advance knowledge*. An example provided by one younger adult exemplifies this:

Curiosity is generally a positive trait....It encourages individuals to...challenge assumptions, leading to deeper understanding and creativity. Curiosity fuels scientific discoveries, technological advancements, and artistic expression, making it essential for progress in various fields. However, curiosity can have negative aspects if it is misdirected or excessive.

In 14.67% of responses, curiosity was described as desirable *in moderation* as it allows individuals to learn new content (i.e., curiosity is *novelty-driven*). This was expressed in the following response: “I do think that it [curiosity] could be both depending on how you use it. It could be positive by being curious about new things to learn.”

The next most prevalent combination was that curiosity is a *positive trait* given its idiosyncratic manifestation within individuals (i.e., *individual differences*). In over a tenth (13.33%) of responses, younger adults described the desirability of curiosity in tandem with how individuals play an active role in its manifestation. For example: “I think it [curiosity] is a positive trait. Because as we grow older I think it is rare for people to have such great curiosity. It is just up to a person on how they execute curiosity they have for the good.”

About a tenth (10.67%) of the sample stated that while curiosity should be expressed *in moderation*, it is a positive trait as it allows for critical thinking (*critical process*). Individuals expressed that curiosity can be both positive and negative, however, it is mainly positive when expressed with a desire to question or seek deeper understanding about something.

I see curiosity as a largely positive trait....pushing people to...ask questions, and seek deeper understanding....However, in some cases, unchecked curiosity can lead to unnecessary risks or distractions. But overall, when balanced with critical thinking and purpose, curiosity is a powerful tool...

Lastly, in 10.67% of responses, younger adults noted that curiosity is a *positive trait* as it is a central aspect of being human (i.e., expressing *centrality*). One participant described curiosity as “positive....I feel our brains are wired to be curious about things and our lives…”

***Older Adult Coding Category Combinations***

In almost half of all responses (45.00%; see S4 Table; S2 Figure) older adults stated that curiosity is a *positive trait* as it allows for *motivated learning*. One older adult wrote: “I strongly feel that it [curiosity] is a positive trait. Curiosity can lead to inspirational moments in life when following wherever it leads one. To me, life without curiosity would seem dull and more monotonous.” Participants expressed a joy in actively engaging in curiosity-driven exploration and viewed this as a positive characteristic.

In almost a third of participants (28.75%), curiosity was described as a *positive trait* given its ability to *advance knowledge*. Put differently, older adults viewed curiosity as a desirable trait as it drives innovation, creativity, and learning in different domains. This is expressed in the following response: “I do think curiosity is a positive trait since, if we were not curious there would be no discoveries in the world. How did we venture into space, discover lifesaving medicines or even, from a young age, learn, read, write, do math etc..”

Less than a quarter (21.25%) of older adults signified curiosity to be a *positive trait*, rationalizing this response in *miscellaneous* ways. Interestingly, while respondents stated that curiosity is desirable, their rationale were in some cases vague: “I would think [curiosity is] very positive otherwise you might as well just sit in the dark and let your life be wasted, and that would be a sad way to spend time, what negative can there be about being curious.”

Similarly, less than a quarter (21.25%) of participants designated curiosity as a *positive trait* given its ability to demonstrate *sincerity* while interacting with others and/or the surrounding environment. For example, one older participant described: “Curiosity is a very positive trait. I feel people who are genuinely curious have closer relationships and are viewed more positively.”

Older adults (20.00%) described curiosity as a *positive trait* due to *individual differences*. Elaborations described idiosyncratic manifestations of curiosity. For instance, one participant wrote a personal description: “Curiosity is a very positive trait. I tend not to ask questions which can limit the depth of relationships.”

Curiosity was also noted as a *positive trait* given its ability to foster a *critical process* (17.50%). Older adults expressed how curiosity is a means by which to question and understand the world around them. For example, one participant indicated: “I see curiosity as a positive trait. It can provide information, solutions and lead to additional questions.”

Older adults (17.50%) also noted curiosity to be a *positive trait* given its ability to promote engagement with novel stimuli (i.e., *novelty-driven*). There was a demonstrated desire to actively engage in and learn new material: “Curiosity is a positive trait. Every experience we have is technically new in some way as we have not lived it before. Curiosity allows us to take advantage to learn about every new experience.”

Older adults (15.00%) noted that curiosity could be positive or negative (i.e., *in moderation*) as it can be *harmful* or lead to disagreeable outcomes. One response stated: “I believe it [curiosity] is situational. But there is a limit and risk to always being curious - it could lead to wasted effort or even potentially very negative outcomes if you are curious too much or about the wrong things.”

In 15.00% of responses from this sample, curiosity was overall seen as a *positive trait*, although how one practices their curiosity could lead to *harmful* situations. Older adults explicitly stated that curiosity is a positive quality, yet they caution that how it is expressed could lead to dangerous circumstances, for example, curiosity is “definitely positive...being curious can sometimes lead to knowing something upsetting, sad, or even dangerous.”

Lastly, 15.00% of older adults indicated curiosity to be a *positive trait* as it promotes *personal growth*. Curiosity can foster individuals to develop their skills and/or behaviours, and can promote a deeper understanding about oneself, which is viewed fondly. One participant noted: “For the most part curiosity is a positive trait that allows one to learn and grow.”
