# Supplementary material for: Age differences in the conceptualization and experience of curiosity: A qualitative study
Source: PLoS One. 2026 May 20;21(5):e0345902. doi: 10.1371/journal.pone.0345902 (PMC13189317; doi:10.1371/journal.pone.0345902)
Supplement: S1 Fig — Note. Frequency counts for top (i.e., appear in ≥ 10% of all responses) combinations of initial and rationale coding category combinations are shown for the younger adult sample, illustrating how and why curiosity is experienced (i.e., in terms of its valence) as either a positive or negative trait, or a trait to be expressed in moderation. (PDF) [file pone.0345902.s004.pdf]

S1 Figure

Frequency Counts for Combinations of Coding Categories in Younger Adults: Valence of Curiosity

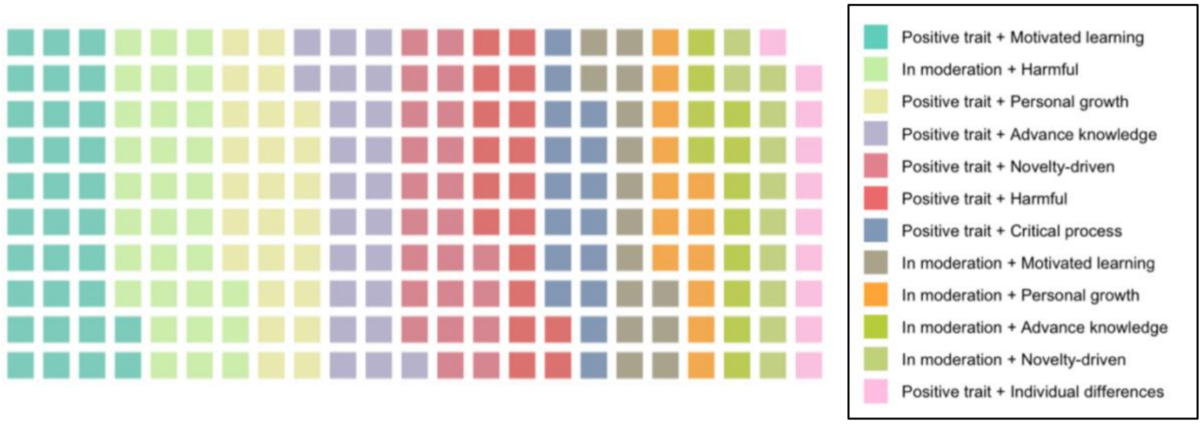

Note. Frequency counts for top (i.e., appear in ≥ 10% of all responses) combinations of initial and rationale coding category combinations are shown for the younger adult sample, illustrating how and why curiosity is experienced (i.e., in terms of its valence) as either a positive or negative trait, or a trait to be expressed in moderation.
