# Supplementary material for: Age differences in the conceptualization and experience of curiosity: A qualitative study
Source: PLoS One. 2026 May 20;21(5):e0345902. doi: 10.1371/journal.pone.0345902 (PMC13189317; doi:10.1371/journal.pone.0345902)
Supplement: S3 Table — Note. Order of frequencies presented in order of most to least frequent. (DOCX) [file pone.0345902.s005.docx]

**S3 Table. Most Frequent Category Code Combinations for Younger Adult Sample.**

| **Categories** | | **Examples** | **Freq.** **%** |
| --- | --- | --- | --- |
| ***Initial Response*** | ***Rationale*** |  |  |
| Positive trait | Motivated learning | "... however, I believe that it [curiosity] is much more positive than negative. Curiosity allows us to not be ignorant about many topics and it allows us to stay informed. There is a famous quote that says 'Ignorance is bliss' which I do not agree with at all. I believe ignorance is a very negative trait that can be combated by curious minds."  "I believe curiosity is a positive , regardless of consequence and outcome. If an activity or subject is entered with a curious state of mind, any mistakes made are learning opportunities."  "I think curiosity is more of a positive trait than a negative. This is because curiosity is what drives us to knowledge throughout our entire lifespan." | 42.67% |
| In moderation | Harmful | "I believe curiosity is such a positive trait....I believe curiosity is negative if its for the wrong reasons. For example listening to the conversations of others to judge them rather than listening to understand them instead."  "[Curiosity is] positive and negative...we tend to go further than we should and putting more money and focus on less relevant things."  "Curiosity is generally a positive trait...However, curiosity can have negative aspects if it is misdirected or excessive. Additionally, an overwhelming sense of curiosity without focus can lead to distractions or unnecessary risks, such as engaging in dangerous activities without considering the consequences." | 41.33% |
| Positive trait | Personal growth | "I personally believe it [curiosity] to be more of a positive trait...as I feel like it also enhances personal growth aswell for individuals to gain a deeper understanding."  "I think curiosity in safe amounts is almost always a positive traits. Curiosity helps us grow and learn about who we are, and our place in the world."  "I think of curiosity as a positive trait, someone acting upon that curiousness will lead to new knowledge that will cause a person to grow." | 33.33% |
| Positive trait | Advance knowledge | "I see curiosity as a largely positive trait....Curiosity helps expand perspectives, solve problems, and adapt to change."  "I think curiosity is an overall positive trait....Curiosity is the reason people have made notable discoveries that improve the lives of people."  "I think, curiosity is more of a positive trait....being curious in a way allows for change and new discoveries to be made." | 30.67% |
| Positive trait | Novelty-driven | "Definitely positive. The more curious you are the willing you are to experience new things as well as learning new things."  "I think curiosity is a positive trait....Having a different and unique outlook on life as they are always willing to learn new things and experience life in a different outlook."  "I think [curiosity is] a positive trait because one can learn alot of new things from just being curious...and that allows them to find newer things one can like and explore."  "I think curiosity is definitely a positive trait....Being curious helps us remain passionate and delve into new interests and passions. Being curious allows us to generate new ideas..." | 30.67% |
| Positive trait | Harmful | "My gut instinct is that curiosity sounds positive...but it absolutely can be negative in some contexts (like prying too much into someone's personal matters, maybe even entering stalker territory."  "I think it [curiosity] is a positive trait....It can have negative connotations to it, but overall in safe situations with knowledge of danger is benefical."  "I think curiosity in safe amounts is almost always a positive traits....Of course, too much of anything can be bad, and too much curiosity can be dangerous." | 24.00% |
| Positive trait | Critical process | "I think that curiosity is a positive trait because without being curious, its hard to understand concepts and ideas. If I want to learn how an ikea shelf is made, I will need to want to know how its built and read the instruction. However, if I am not curious, I will not have motivation therefore making it likely that I dont want to build the shelf anymore."  "I think of curiosity as a primarily positive trait....It encourages individuals to ask questions..."  "Curiosity is a positive trait....Imagine if nobody was curious, our world would be dim with no answers to questions and nothing to strive for. " | 21.33% |
| In moderation | Motivated learning | "I think curiosity can definitely be both positive and negative....Curiosity allows us to not be ignorant about many topics and it allows us to stay informed. There is a famous quote that says 'Ignorance is bliss' which I do not agree with at all. I believe ignorance is a very negative trait that can be combated by curious minds."  "I think curiosity can be both positive and negative traits. Being curious is good in the sense [that] you are interested in obtaining further information."  "[Curiosity is] positive and negative....Humans are definitely the most curious species in the world, whether it be space or the ocean or caves, we tend to go further than we should...simply for gaining knowledge..."  "I think [curiosity is] more positive than negative, as it causes a person to want to learn something....However it can have some negative aspects..." | 20.00% |
| In moderation | Personal growth | "I see curiosity as a largely positive trait....However, in some cases, unchecked curiosity can lead to unnecessary risks or distractions. But overall, when balanced with critical thinking and purpose, curiosity is a powerful tool for personal and intellectual growth."  "That [curiosity's valence] depends on the persons self awareness. A person needs to know boundaries and respect them....[curiosity] allows...self growth..."  "I think curiosity can be both positive and negative traits....curiosity in workspaces can lead to excelling in positions." | 17.33% |
| In moderation | Advance knowledge | "I see curiosity as a largely positive trait. It drives...innovation....Curiosity helps expand perspectives, solve problems, and adapt to change. However, in some cases, unchecked curiosity can lead to unnecessary risks or distractions. But overall, when balanced with critical thinking and purpose, curiosity is a powerful tool for...intellectual growth."  "That [curiosity's valence] depends on the person's self awareness. A person needs to know boundaries and respect them....Education wise being curious is great, it allows more knowledge..."  "[Curiosity is] positive and negative, as curiosity is key to innovation..."  "I think [curiosity is] more positive than negative....It fosters problem solving and innovation. However it can have some negative aspects..."  "I think it [curiosity] is both equally positive and negative depending on the context. For example if someone is curious about subjects that will have a benefit in their lives and the lives of those around them, then I do agree that it is good to be curious." | 16.00% |
| In moderation | Novelty-driven | "I do think that it [curiosity] could be both depending on how you use it. It could be positive by being curious about new things to learn."  "I believe [curiosity is] neither but rather dependant on the person...a person who has a love for life, nature, and exploring's curiosity is safer and a positive trait."  "Curiosity is generally a positive trait...However, curiosity can have negative aspects...Overall, when balanced with good judgment, curiosity is a valuable trait that encourages exploration...and new opportunities." | 14.67% |
| Positive trait | Individual differences | "I don't think curiosity could ever be a negative trait. Using your brain and intellect to its highest potential is a great thing."  "It [curiosity] is definitely a positive trait. There is nothing wrong with having a sense of curiosity. All humans behave, look, and speak differently and that is the reason for curiosity."  "I think curiosity is a more positive trait....Being curious can help...provide motivation when it comes to working, school, and everyday life."  "I believe that curiosity is a very beneficial trait! I believe that if you aren't making decisions on your own and questioning things you experience and are told you are living a sort of mindless life. If your not being curious you are going to have less free though, as your thoughts will just be made of a mesh of other peoples thoughts."  "I think of curiosity as a more positive trait. I view it this way because, coming a very curious person in general and someone who thirsts to learn more on a day to day basis, I see curiousity as something that helps people learn more on a day to day basis..." | 13.33% |
| In moderation | Critical process | "Curiosity is generally a positive trait....It encourages individuals to ask questions....However, curiosity can have negative aspects..."  "Curiosity is generally a good thing....It drives...critical thinking, enabling individuals to adjust and broaden their understanding. On the flip side, it can have a downside if it results in invasive behaviour..."  "I think [curiosity is] a positive trait because one can learn...how things work...[but] for example if someone is curious about bombs than it there [*cuoristy*] might be a bad thing." | 10.67% |
| Positive trait | Centrality | "Curiosity is a more positive trait because it helps you to...find meaning in your life."  "I think it [curiosity] is a positive trait...I believe it can bypass many signals in our brains that may have made us choose otherwise."  "I believe [curiosity is] a positive trait....Learning is essentially for us as a species..."  "I think that curiousity is an incredibly positive trait. What's the point of living if you're not curious about anything?"  "It [curiosity] is definitely a positive trait. There is nothing wrong with having a sense of curiosity....We simply cannot help it."  "I think of curiosity as a more positive trait...[curiosity] helps people learn more on a day to day basis and helps them throughout their life and in the world. Without curiosity what is the point in living." | 10.67% |

*Note.* Order of frequencies presented in order of most to least frequent.
