# Supplementary material for: Age differences in the conceptualization and experience of curiosity: A qualitative study
Source: PLoS One. 2026 May 20;21(5):e0345902. doi: 10.1371/journal.pone.0345902 (PMC13189317; doi:10.1371/journal.pone.0345902)
Supplement: S4 Table — Note. Order of frequencies presented in order of most to least frequent. (DOCX) [file pone.0345902.s007.docx]

**S4 Table. Most Frequent Category Code Combinations for Older Adult Sample.**

| **Categories** | | **Examples** | **Freq.** **%** |
| --- | --- | --- | --- |
| ***Initial Response*** | ***Rationale*** |  |  |
| Positive trait | Motivated learning | "It [curiosity] is positive, because one is energized by being confronted with something new, or another viewpoint, and wanting to explore what it all means. It is a way of feeling youthful and open to life..."  "I strongly feel that it [curiosity] is a positive trait. Curiosity can lead to inspirational moments in life when following wherever it leads one. To me, life without curiosity would seem dull and more monotonous."  "Definitely positive. Why would I consider it a negative trait if it is something that I enjoy and do?" | 45.00% |
| Positive trait | Advance knowledge | "I strongly feel that it [curiosity] is a positive trait...helps to find ways to learn about and perhaps contribute in a positive way to our planet and its people."  "I do think curiosity is a positive trait since, if we were not curious there would be no discoveries in the world. How did we venture into space, discover lifesaving medicines or even, from a young age, learn read, write, do math etc.."  "Curiosity is totally positive. Curiosity leads to education or knowledge...any time you can expand or enhance your existing knowledge base is positive from an educational and emotional perspective." | 28.75% |
| Positive trait | Miscellaneous | "I think of curiosity as a positive trait in a persons health and well being lifestyle."  "[Curiosity is] [*definitely*] a positive trait. You would almost be a vegetable if you were not curious."  "I would think [curiosity is] very positive otherwise you might as well just sit in the dark and let your life be wasted, and that would be a sad way to spend time, what negative can there be about being curious." | 21.25% |
| Positive trait | Sincerity | "Curiosity is a very positive trait. I feel people who are genuinely curious have closer relationships and are viewed more positively."  "[Curiosity is] [*definitely*] a positive trait. I can't imagine not being curious, since it shows a lack of interest in almost everything."  "I see it [curiosity] as a positive trait...and creates human connections with others often that allow understanding to develop." | 21.25% |
| Positive trait | Individual differences | "Curiosity is a very positive trait. I tend not to ask questions which can limit the depth of relationships."    "I think it [curiosity] is a hugely positive trait and enriches one's life. It is hard for me to imagine what my life would be like if I were not curious about the things around me."  "I think of curiosity as a positive trait. I think that some people are innately intelligent and are book smart but I believe that some people who are not necessarily well educated but are curious can be as intelligent (not in a book smart degree way) but in a life intelligence way." | 20.00% |
| Positive trait | Critical process | "I see curiosity as a positive trait. It can provide information, solutions and lead to additional questions."  "[Curiosity is] definitely positive. One need to learn to assess, evaluate and direct how one approaches that which is curious."  "Curiosity is a positive trait. It can help solve problems." | 17.50% |
| Positive trait | Novelty-driven | "I think it [curiosity] is positive...Finding out about new things to do, see and experience is fun."  "Curiosity is a positive trait. Every experience we have is technically new in some way as we have not lived it before. Curiosity allows us to take advantage to learn about every new experience."  "I think it [curiosity] is positive...It allows me to pursue avenues of thought that lead me to learning new things to any degree or extent that I want or need to to achieve a better understanding of any topic." | 17.50% |
| In moderation | Harmful | "I think that curiosity is always positive but some of ones friends may find it a negative - an irritant! Therefore, one has to sometimes restrain oneself from exploring all available avenues and be content to meekly accept the common concept. This can be frustrating!"  "[Curiosity is] positive. It could be a negative trait if you become obsessive about the subject."  "I believe it [curiosity] is situational. But there is a limit and risk to always being curious - it could lead to wasted effort or even potentially very negative outcomes if you are curious too much or about the wrong things." | 15.00% |
| Positive trait | Harmful | "[Curiosity is] definitely positive...being curious can sometimes lead to knowing something upsetting, sad, or even dangerous."  "[Curiosity is] positive. It could be a negative trait if you become obsessive about the subject."  "Definitely positive. That does not mean that there may not be negative results. It needs to be controlled." | 15.00% |
| Positive trait | Personal growth | "I think it [curiosity] is positive...to better understand myself..."  "Curiosity is definitely a positive trait. As individuals, personal growth requires curiosity."  "For the most part curiosity is a positive trait that allows one to learn and grow." | 15.00% |

*Note.* Order of frequencies presented in order of most to least frequent.
