# Supplementary material for: Age differences in the conceptualization and experience of curiosity: A qualitative study
Source: PLoS One. 2026 May 20;21(5):e0345902. doi: 10.1371/journal.pone.0345902 (PMC13189317; doi:10.1371/journal.pone.0345902)
Supplement: Attachment — Submitted filename: Response to Reviewers.pdf [file pone.0345902.s009.pdf]

Thank you for your helpful feedback and suggestions, they have made our manuscript stronger. We have addressed all concerns and are submitting revised versions of the manuscript with and without track changes. Please find our response to the reviewers below:

Reviewer #1:

Weaknesses: Potential priming bias: Quantitative curiosity scales administered before open-ended questions may influence responses, despite miscellaneous codes mitigating this. Limited generalizability due to demographics—primarily female samples, older adults mostly White, convenience sampling from York University pools. Exploratory design lacks quantitative depth; chi-squares are descriptive but not adjusted for multiple comparisons.

Thank you for pointing out that chi-squares were not adjusted for multiple comparisons. We have now included all fdr-adjusted p-values for all tests conducted (S1 Table, S2 Table).

Recommendation: Expand diversity in future work, clarify priming impacts, and provide supplementary code frequencies (e.g., S1/S2 Tables). Strong contribution to developmental psychology of curiosity.

Thank you for these helpful recommendations. In the revised manuscript (page 35-36), we have expanded the Discussion to further emphasize the importance of diversity in future work and to clarify the potential impacts of priming. Code frequencies for younger and older samples are reported within the subheadings of each coding category. In addition, results from the S1 and S2 tables are reported in the main text where significant age differences were observed.

Reviewer #2:

I had the opportunity to review the manuscript titled "Age differences in the conceptualization and experience of curiosity: A qualitative study". The manuscript addresses the important topic of curiosity. However, it attempts to take an approach different from standard curiosity research that is typically quantitative by employing qualitative methods. While I appreciate the effort of the researchers, I think the study falls short of a rigorous and insightful qualitative study that would provide deep insights. The main data is essentially open ended responses to a survey that is mostly quantitative using 5DC and InC items. And a major flaw is that these items are presented first before the open-ended responses, which leads to all sorts of problems as the authors acknowledge. And more importantly, I would expect qualitative research to provide deeper insights into participants' responses via more proving prompts or follow-up interviews, for example. Instead, the respondent data is mostly quite superficial from a paid survey where the researchers had to enforce a minimum response length. Questions such as why the

respondents said what they said, why they did not say certain things, how their views on curiosity may have changed over time (which is one way to address the question of curiosity differing by age), etc. are all left unaddressed. The authors seemed to have collected quantitative data from 5DC and InC but chose not to report them here (or triangulate with the qualitative data) (perhaps, saving for another publication?). There have been other studies that have employed qualitative approaches on curiosity (including mixed methods) with much more methodological rigor that I would have expected this manuscript to at least match. They used more probing prompts, interviews, combined quantitative data, etc. to obtain, in my opinion, deeper insights. Below are some examples:

Han, J., Way, N., Yoshikawa, H., & Clarke, C. (2025). Interpersonal curiosity and its association with social and emotional skills and well-being during adolescence. Journal of Adolescent Research, 40(3), 636-668.

Birenbaum, M., Alhija, F. N. A., Shilton, H., Kimron, H., Shahor, N., & Rosanski, R. (2023). Curious minds: Evidence from interviews with renowned experts in five curiosity-dominant fields.

Birenbaum, M., Alhija, F. N. A., Shilton, H., Kimron, H., Rosanski, R., & Shahor, N. (2019). A further look at the five-dimensional curiosity construct. Personality and Individual Differences, 149, 57-65.

We thank the reviewer for their thoughtful concerns about our qualitative design. We agree that qualitative approaches that allow for probing and iterative clarification can provide richer insight into participants' reasoning and lived experiences. In the present study, however, our goal was to conduct structured content analysis of responses to questions about lay conceptualizations of curiosity among younger and older adults, rather than in-depth phenomenological interviews (like Birenbaum et al., 2023). Our online design prioritized breadth of responses over the depth of individual conceptualizations. While this approach limits our ability to fully explore participants' response motivations (e.g., why participants did or did not say certain things, and how their views on curiosity may have changed over time), it enables us to characterize overall patterns in how younger and older adults conceptualize curiosity using common prompts. Accordingly, our findings should be interpreted as providing an initial descriptive overview rather than an in-depth phenomenological account. To our knowledge, age differences in how people define curiosity have not been directly examined, and we position our study as a first exploratory step. We now expand on this (page 36), noting that future works employing more intensive qualitative methods may be better-suited to further understand how and why lay conceptualizations of curiosity differ as a function of age.

Additionally, as you pointed out, participants completed the 5DC and InC scale items. These measures were included to address separate research questions, which are reported elsewhere (p. 8). We agree that carryover effects from completing the 5DC and InC items represent a

limitation. In the revised manuscript (pp. 35–36), we expand our discussion of this issue and clarify that the primary aim of the present study was to examine age differences in how curiosity is conceptualized, rather than to derive novel dimensions of curiosity. While completing the scale items may have influenced the overall content of responses, it is less clear that such priming would systematically differ between younger and older adults. As such, the observed differences between age groups in coding categories remain informative with respect to our primary research question, although we cannot definitively rule out the influence of priming on participants' responses. Importantly, over half of responses were coded as miscellaneous, indicating that participants frequently articulated themes that extended beyond the dimensions captured by the 5DC and InC measures.

I also question some of the claims made about 5DC and InC not able to capture some participant responses such as individual differences and innovation. The point of 5DC is to measure individual differences, cluster, and build curiosity profiles of individuals. And innovation partly overlaps with novelty aspects of I-type curiosity by Litman.

Thank you for pointing this out. Indeed, the 5DC is designed to capture individual differences (in addition to clustering and building curiosity profiles), and innovation partly overlaps with novelty aspects of Litman's I-type curiosity. That said, items from the 5DC and InC were collated to serve as code definitions guiding the deductive coding of participant responses to Question 1. Although the innovation code relates to Litman's I-type curiosity, and individual differences are captured when participants respond directly to the 5DC scale, our coding scheme focused on mapping open-ended responses onto predefined conceptual categories. Responses that did not align with these predefined codes were therefore assigned to separate categories rather than being subsumed under 5DC-derived codes (e.g., innovation, individual differences).

It's unclear what the unit of analysis is for the code. It's clear that some participant responses are long and cover different aspects. I assume those responses are being given multiple codes? But some examples given are very short with ellipses, so I assume they've been trimmed by the authors?

While responses were required to have a minimum of 250 characters, response lengths varied across participants. The unit of analysis for coding was the full participant response. Accordingly, multiple codes were assigned to a single response when it reflected more than one coding category. We have now included additional clarification on how codes were assigned (pages 9-10).

For descriptive purposes, some responses were trimmed by the authors and presented as code exemplars, which may result in shorter excerpts with ellipses. We have now also provided a more comprehensive dataset (osf.io/vqxfr) containing full participant responses, where consent for data sharing was provided.

I don’t see how refs 1 and 2 can be used to support the statement “seen a resurgence in research on human curiosity”.

Thank you for pointing this out. You are correct that References 1 and 2 did not appropriately support the statement regarding a “resurgence in research on human curiosity.” This was an error on our part. In the revised manuscript, we have replaced these citations with more appropriate and up-to-date references that directly support this claim.

Consider a more recent reference in PLoS One that can replace current ref 15: https://doi.org/10.1371/journal.pone.0320600

Thank you for this recommendation. We have now replaced the current ref 15 with Whatley et al. (2025; page 4).

Reviewer #3:

My first question is about the fact that participants' "initial responses" to the valence question were coded. It's not entirely clear what the "initial response" is - is that the first few words that address the issue of valence? Was there a clear cutoff for initial response vs. rationale? Additionally, were there any cases where the rationale was overall different from the initial response? (e.g., they said it was positive but discussed negative aspects throughout the response)

For the valence question, the initial response was defined as the participant’s classification of curiosity as positive, negative, or something to be expressed in moderation. This represents the “what” component of the response. In contrast, rationales were defined as any part of the response that followed this initial classification, reflecting the “why” underlying the participant’s judgment.

For example, if a participant responded, “Positive because it allows me to learn more about myself,” the response would be coded as positive for the initial code category, and personal growth for the rationale code category. We have now included additional clarification on how initial and rationale codes were assigned (pages 9-10).

Because rationales were not inherently positive or negative in valence, instances of mismatch between initial classifications and rationale codes were rare.

It's not clear how the categories were decided on for the post-hoc coding into motivated learning, critical process, etc. Was there any specific process for coming up with these categories? And could the responses also count in more than one of these categories?

Thank you for bringing up this point of confusion. For the post-hoc coding of the valence question, the process was as follows. Briefly, two primary coders independently reviewed 50% of the responses. Following this initial review, each author began grouping responses into coding categories based on observed patterns, iteratively developing and refining category names based on the data. After completing this step, the authors met to discuss and reconcile category definitions, adjusting category names and descriptions until consensus was reached. Following this, the coding scheme was applied to the full dataset, with responses assigned to initial and rationale coding categories. Coding consistency was assessed by an additional coder on a subset of responses, with discrepancies resolved through discussion.

Responses could be assigned to more than one category when they reflected multiple themes. We have now clarified both the coding procedure and the use of multiple coding in the manuscript (pages 9-10).

Similarly, it's interesting that morbid curiosity is a somewhat well-known phenomenon and these ideas tended to come up (e.g., the person who said they always ask how people died because they are interested, even if it's considered rude!). Did the authors consider including a morbid curiosity category for responses? The discussion of the potential for this category to be accounted for in measures of curiosity could also be expanded.

This is a very interesting point. As the valence question was coded inductively (i.e., post-hoc), coding categories were derived directly from participants' responses rather than being guided by existing theoretical frameworks (see response to above comment). Accordingly, we did not include a predefined morbid curiosity category during the coding process.

That said, we agree that some responses—such as those reflecting interest in death or other negatively valenced content—are conceptually related to morbid curiosity. We have now expanded the Discussion (pages 35-36) to highlight this connection and its implications for exploring age differences in the experience of curiosity.

Were the responses of similar length across age groups? It seems like this was conducted on the computer, and older adults may be less accustomed to computers (though this is less of a concern as time passes). Is it possible that one age group expanded on their ideas more than another?

Responses appeared to vary more across individuals than between age groups. We have now provided a more comprehensive dataset (osf.io/vqxfr) containing full participant responses, where consent for data sharing was provided.

Could you say more about how older adults were recruited? I do not know what the Glendon Research Participant Pool is - are these participants involved in the university setting?

Certainly! The Glendon Research Participant Pool consists of community dwelling older adults that consented to be contacted about studies conducted at Glendon College. These participants are not involved in the university setting. We have now included further clarification on this (page 6).

I think some of the references are off - especially in the 9-15 range.

Thank you for pointing this error out. We have now updated the references accordingly.
